# The Development of Infants’ Use of Novel Verbal Information when Reasoning about Others' Actions

**DOI:** 10.1371/journal.pone.0092387

**Published:** 2014-03-24

**Authors:** Hyun-joo Song, Renée Baillargeon, Cynthia Fisher

**Affiliations:** 1 Department of Psychology, Yonsei University, Seoul, Korea; 2 Department of Psychology, University of Illinois, Champaign, Illinois, United States of America; Queen Mary University of London & University College London, United Kingdom

## Abstract

How sophisticated are infants at using novel verbal information when reasoning about which of two objects an agent is likely to select? The present research examined the development of infants’ ability to interpret a change from one novel word to another as signaling a possible change in which object the agent would choose next. In three experiments, 7- and 12-month-olds were familiarized to an event in which they heard a novel word ("A dax!") and then saw an agent reach for one of two distinct objects. During test, the infants heard a different novel word ("A pilk!") and then saw the agent grasp the same object or the other object. At 7 months, infants ignored the change in word and expected the agent to continue reaching for the same object. At 12 months, however, infants attended to the change in word: They realized that it signaled a possible change in the agent’s upcoming actions, though they were unable to form a specific expectation about what these new actions might be, most likely due to their limited mutual-exclusivity assumption. Control conditions supported these interpretations. Together, these results suggest that by 12 months of age, infants understand not only that words are selected for communicative purposes, but also that a change from one novel word to another may signal a change in an agent’s upcoming actions.

## Introduction

As adults, we can use several different types of information to interpret and predict others’ actions. For example, if an agent was facing two different objects and we were attempting to predict which object the agent was likely to select, we might consider (1) what prior choices the agent had made given the same two objects to choose from, (2) what emotional expressions the agent had displayed toward the objects, and (3) what verbal communications the agent had spoken or heard concerning the objects. Thus, if we were amusing ourselves at a family reunion by trying to predict which of two desserts, chocolate cake or pecan pie, our relatives were likely to choose at the buffet table, we might predict that (1) Uncle Albert would prefer the cake, because he had chosen it on his three previous turns at the buffet table, (2) Aunt Edna would opt for the pie, because her face had lit up when she noticed it, and (3) Cousin Harry would also choose the pie, because he had loudly announced “No more lasagna, I’m ready for some pie!” as he headed toward the buffet table.

Research over the past 15 years indicates that infants, too, can form expectations about which object an agent is likely to select in a two-object situation [1]–[17]. By about 12 months of age, as we review below, infants can use all three types of information discussed above—prior-choice, emotional, and verbal information—to form these expectations. The present research built on these findings and examined how sophisticated is infants’ ability to use verbal information—and particularly *changes* in verbal information—when reasoning about an agent’s actions.

### Prior findings

Beginning with Woodward’s seminal research [10], numerous experiments have shown that infants attend to *prior-choice information* in a two-object situation to determine which object the agent is likely to act on next [1], [2], [7], [10], [11], [15], [16], [18], [19]. To illustrate, Guajardo and Woodward [2] habituated 12-month-olds to an event in which two distinct objects, a teddy bear and a ball, rested on opposite sides of an apparatus floor; in each habituation trial, a female agent reached for and grasped the same object (e.g., the bear; henceforth the target object). Following habituation, the objects’ positions were switched, and the infants saw two test events. In the old-object event, the agent reached for the target object in its new position; in the new-object event, she reached for the non-target object. The infants looked reliably longer at the new- than at the old-object event, suggesting that they (1) attributed a preference for the target object to the agent, based on her unvarying choices across the habituation trials, (2) expected her to continue acting on this preference in the test trials, and hence (3) detected a violation when she reached for the non-target object instead. Subsequent findings supported this interpretation and ruled out deflationary interpretations based on associative learning or low-level novelty. In particular, infants did not attribute a preference for the target object to the agent when the non-target object was absent in the habituation trials [4], [20]. Although the agent reached for the target object as before, her actions no longer provided choice information; as a result, infants had no basis for determining her disposition toward the target object, and they tended to look equally in the test trials whether she reached for the target or the non-target object.

When the target and non-target objects in a two-object situation belong to the same taxonomic category (e.g., two dolls), 12-month-olds do not attribute to the agent a preference for the target object [15], suggesting that they tend to encode agents’ preferences in categorical terms (e.g., a preference for dolls). Nevertheless, infants can use *emotional information* to predict which of the two objects the agent is likely to act on next. For example, Phillips et al. [6] habituated 12-month-olds to an event in which a female agent first looked at one of two toy cats with a facial and vocal expression of joy; next, a curtain was drawn in front of the scene and then re-opened to reveal the agent holding that same cat. Following habituation, the infants watched two test events. In the consistent event, the agent first emoted positively toward the other cat; when the curtain was re-opened, she held that cat. In the inconsistent event, the agent first emoted positively toward the same cat as in the habituation trials; when the curtain was re-opened, however, she again held the other cat. The infants looked reliably longer at the inconsistent than at the consistent event, suggesting that they considered the agent’s gaze and emotional expression to determine which of the two cats she would hold next.

Recent work by Martin, Onishi, and Vouloumanos [5] indicates that a third type of information 12-month-olds can use in two-object situations is *verbal information.* This work built on prior findings that by 9 months infants already perceive words as linguistic conventions that are shared and understood by members of the same speech community [1], [21], [22]. Martin et al. asked whether 12-month-olds (1) would interpret an agent’s novel word as referring to her preferred (as opposed to non-preferred) object, even without any prior pairing of the word and preferred object, and (2) would expect another agent who heard the novel word to understand its referent. The infants first received three familiarization trials in which agent1 sat at a large window in the back wall of an apparatus, behind two distinct objects. In each trial, agent1 picked up the same (target) object and tilted it back and forth until the trial ended; her unvarying choices signaled that she had a preference for the target object. Next, the infants received a pretest trial in which agent1 was absent (her window was closed); agent2 sat at a window in the right wall of the apparatus and faced the same two objects as in the familiarization trials. Agent2 picked up and tilted each object in turn, indicating that she had no preference for either object. Finally, the infants received a single test trial in which agent1 now peered through a small window in the back wall of the apparatus and thus no longer had access to the objects; agent2 remained present and could still reach for both objects. Agent1 looked at agent2 and said, “Koba! Koba!” Agent2 then picked up either the target (old-object event) or the non-target (new-object event) object and lifted it just below agent1’s window, as though presenting her with the object. The infants who saw the new-object event looked reliably longer than those who saw the old-object event. This and control results suggested that the infants (1) assumed that agent1’s communication specifically referred to the target object (since agent2 was absent during the familiarization trials, the word “koba” had to convey sufficient information to identify the target object), (2) expected agent2 to know the referent of the word “koba”, and hence (3) detected a violation when agent2 picked up the non-target as opposed to the target object. These results thus provided evidence that by 12 months of age, infants can already use verbal information to predict which of two objects an agent is likely to act on next.

### The present research

The results reviewed in the previous section suggest that 12-month-olds can predict which of two objects an agent will select in a scene based on (1) prior-choice information indicating which object (or which kind of object) the agent prefers and (2) a relevant communication by the agent or another party in the scene. The present research built on these results and asked whether 12-month-olds could interpret a *change* in communication as signaling a possible *change* in the agent’s upcoming actions.

**Figure 1:**
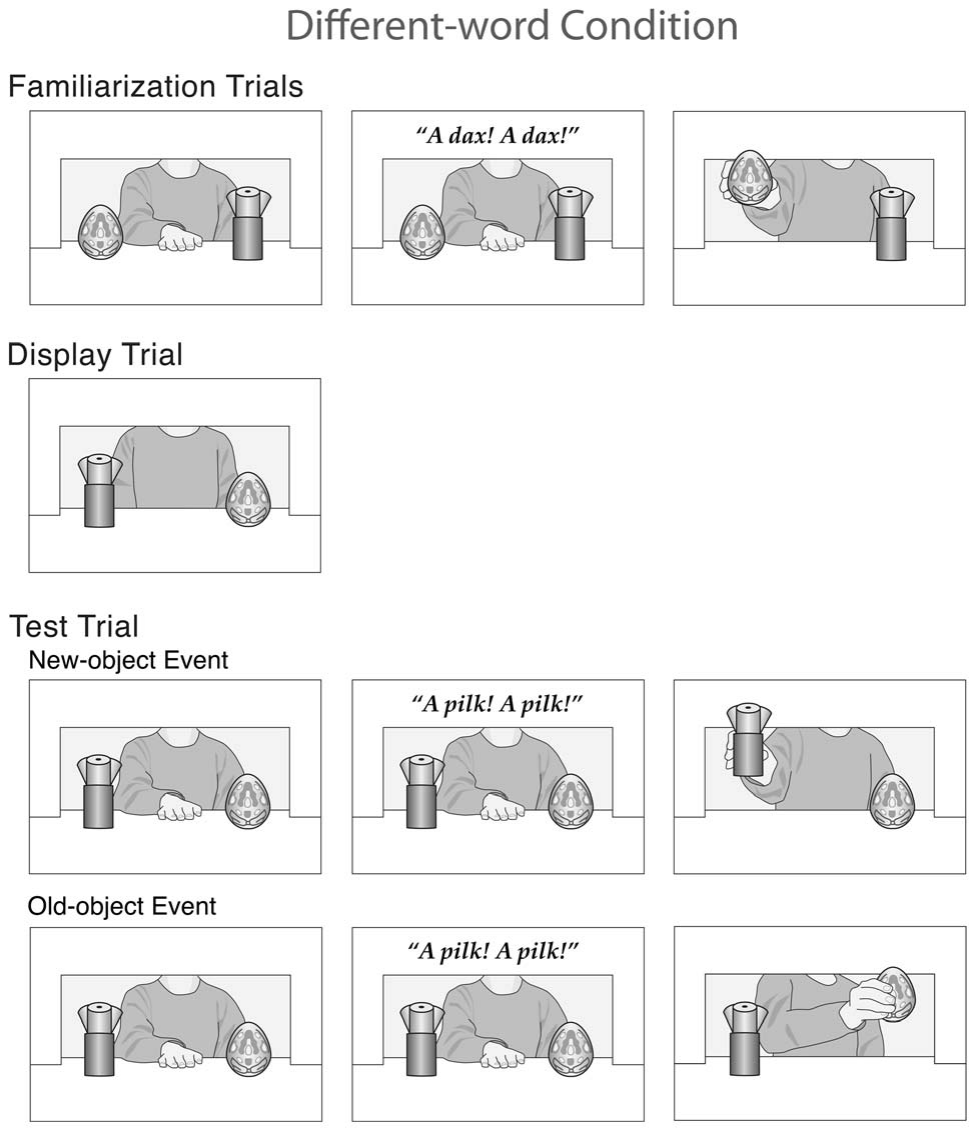
Schematic drawing of the events presented in the familiarization, display, and test trials of the different-word condition in Experiment 1.

Infants were tested in a no-word, same-word, or different-word condition. The *no-word* condition was adapted from Guajardo and Woodward’s [2] preference task and included four familiarization trials, one display trial, and one test trial. During the familiarization trials, a female agent sat behind two distinct objects (Fig. 1) and reached for the same (target) object across trials. During the display trial, the objects’ positions were switched. Finally, during the test trial, the agent reached for either the target object in its new position (old-object event) or the non-target object (new-object event). We predicted that, as in previous preference tasks, the infants would attribute to the agent a preference for the target object and hence would look reliably longer if shown the new- as opposed to the old-object event.

The *same-word* condition was identical to the no-word condition except that at the start of each trial, before the agent reached, the infants heard the novel word “A dax!” spoken twice in a female voice. If the infants interpreted this word as referring to the target object and announcing the agent’s intention of reaching for that object, then they should detect a violation in the new-object event, because the agent reached for the non-target object even though the same word was heard at the start of the trial.

Of most interest for present purposes was the *different-word* condition, which was identical to the same-word condition except that a different novel word, “A pilk!”, was heard at the start of the test trial. We reasoned that there were at least two ways the infants might respond. One possibility was that the infants would notice the change in word at the start of the test trial, assume that this new word referred to the non-target object, and form the expectation that the agent would now reach for this object; the infants should thus look reliably longer if shown the old- as opposed to the new-object event (the opposite pattern from that predicted for the no-word and same-word conditions). Another possibility was that the infants would detect the change in word at the start of the test trial and realize that this change might signal a change in the agent’s upcoming actions, but be unable to form a specific expectation about what these new actions might be. In this case, the infants should look about equally at the new- and old-object events.

To adults operating on a full-fledged mutual-exclusivity assumption (i.e., in many contexts, different words refer to different objects; [23]), the first possibility discussed above might seem by far the more likely; after all, what else could “A pilk!” refer to but the non-target object, the sole other object present on the apparatus floor? However, prior research on the development of the mutual-exclusivity assumption suggested that what might appear trivially obvious to adults might not be so for 12-month-old infants. This prior research can be summarized in terms of three main findings. First, 9- to 12-month-olds can use distinct novel words to individuate objects; for example, if an experimenter produces two different words while peering into a box, infants conclude that at least two objects are in the box [24], [25]. Second, when faced with a familiar object (whose label is known) and a novel object, infants as young as 17–18 months will spontaneously link a novel word to the novel object [26]–[28]. Third, after being taught a novel word for a novel object, infants age 18 months and older will spontaneously link a different novel word to a different novel object [29]–[31]. Together, these findings suggest that although 12-month-olds possess a fledgling assumption of mutual exclusivity (and, in particular, expect two distinct objects upon hearing two distinct words), they struggle to use this assumption to interpret new words. Therefore, it seemed possible that the infants in the different-word condition might show only *partial* understanding in their responses: They might realize that the change in word at the start of the test trial communicated a possible change in the agent’s actions, but be unable to form a specific expectation about what these new actions might be.

## Experiment 1

### Methods

#### Participants

Participants were 46 healthy term infants, 24 male and 22 female (11 months, 7 days to 12 months, 29 days, *M* = 12 months, 3 days). Sixteen infants were randomly assigned to the different-word condition (*M* = 12 months, 1 day), 14 infants were randomly assigned to the same-word condition (*M* = 12 months, 6 days), and 16 infants were randomly assigned to the no-word condition (*M* = 12 months, 3 days). Half the infants in each condition saw the new-object event during the test trial, and half saw the old-object event. Another 17 infants were tested but excluded because they looked for the maximum amount of time allowed (40 s) in the test trial (9), were inattentive (4), fussy (2) or distracted (1), or had a looking time in the test trial over 2.5 standard deviations from the mean of the condition (1).

The infants’ names in this and the subsequent experiments were obtained primarily from purchased mailing lists and from birth announcements in the local newspaper. Parents were offered reimbursement for their travel expenses but were not compensated for their participation. Each infant’s parent gave written informed consent, and the protocol was approved by the Institutional Review Board at the University of Illinois at Urbana-Champaign.

#### Apparatus

The apparatus consisted of a wooden display booth (98 cm high, 100 cm wide, and 50 cm deep), mounted 76 cm above the floor. The infant faced a large opening (42 cm×93.5 cm) in the front wall of the apparatus; between trials, an experimenter lowered a muslin curtain (61 cm×99.5 cm) in front of this opening. The floor of the apparatus was covered with pastel adhesive paper and the side walls were painted white. The back wall was made of white foam core; a window (20 cm×54 cm) extended from its lower edge, 6 cm from the right wall.

The agent sat on a wooden chair centered behind the window. As in many previous preference tasks [32], [33], the agent’s head was hidden from the infants’ view; only her arms and torso were visible, covered in a blue shirt. A muslin screen behind the agent hid the testing room. To the left of the window, behind the back wall of the apparatus, were two speakers and a CD player. The CD player was activated by the experimenter and was used to play the words heard in the different- and same-word conditions. To ensure similarity across infants (as the agent’s head was not visible), the words played during the familiarization and test trials were digitally recorded on a CD by a female native speaker of English. The two tokens of each word were identical and played at a comfortable listening level (about 68 dB, measured with a hand-held sound level meter).

The two objects used in each trial were a toy egg and a toy tree; they were highly dissimilar and hence were likely to be perceived as belonging to distinct object categories [15]. The two objects were positioned 22 cm in front of the window, 13.5 cm from the window’s left and right edges. In the familiarization trials, the egg was on the left and the tree was on the right; in the display and test trials, these positions were switched. The egg (8.5 cm×6.5 cm in diameter) was made of turquoise plastic and was decorated with a multi-colored turtle design. The tree (10.5 cm×5 cm in diameter) was made of plastic and consisted of a red cylindrical base (6 cm×4 cm in diameter) from which sprouted three purple “branches” (each 3.5 cm×3 cm in diameter) with yellow, orange, and turquoise tops.

The infants were tested in a brightly lit testing room. Three 20-W fluorescent light bulbs were attached to the front and back walls of the apparatus to provide additional light. Two frames (each 180.5 cm×9.5 cm and covered with white cloth) stood at an angle on either side of the apparatus; these frames served to isolate the infants from the testing room.

#### Procedure

The infant sat on a parent’s lap centered in front of the apparatus; the infant’s head was approximately 53 cm from the curtain. Parents were instructed to close their eyes and to remain silent and neutral during the testing session.

The infants in the *different-word* condition received four familiarization trials, one display trial, and one test trial (in the following descriptions, the numbers in parentheses indicate the number of seconds the agent took to perform the actions described). Each *familiarization* trial had an initial phase and a final phase. At the start of the 6-s initial phase, the agent sat at the window with her bare right hand resting on the apparatus floor, centered between and 15 cm behind the egg and tree. After a 2-s silent pause, a novel word (“A dax!”) was played twice from the two speakers located behind the back wall (2 s). Next, the agent reached for and grasped the egg (1 s), and then lifted it about 10 cm above the apparatus floor (1 s). During the final phase of the trial, the agent tilted the object gently to the left and right, changing orientation once per second, until the trial ended. The duration of the final phase was determined by the infant’s looking behavior, as described below.

Prior to the *display* trial, the toys’ positions were switched. During the trial, the infants saw the toys in their new positions; the agent sat at the window, with no hand on the apparatus floor. This trial was static and had only a final phase.

Finally, the *test* trial again had an initial and a final phase. At the start of the 6-s initial phase, the toys were in the same switched positions as in the display trial, and the agent sat with her hand on the apparatus floor. After a 2-s silent pause, a different novel word (“A pilk!”) was played twice (2 s), and then the agent reached for, grasped, and lifted either the egg (old-object event) or the tree (new-object event) (2 s). During the final phase of the trial, the agent tilted the toy back and forth until the trial ended, as in the familiarization trials.

The trials in the *same-word* and *no-word* conditions were identical to those in the different-word condition with the following exceptions. In the *same-word* condition, the same word (“A dax!”) was played during the initial phase at the start of each familiarization and test trial. In the *no-word* condition, no word was played during the initial phases of the familiarization and test trials; each initial phase thus began with a 4-s, instead of a 2-s, silent pause.

To help the agent adhere to the events' scripts, a metronome beat softly once per second. A camera mounted behind and next to the infant projected an image of the events onto a TV screen in a different part of the testing room; a supervisor monitored the events to confirm that they followed the prescribed scripts

The infant's looking behavior was monitored by two observers who viewed the infant through peepholes in the cloth-covered frames on either side of the apparatus. The observers could not see the events from their viewpoints. Each observer held a button linked to a computer and depressed the button when the infant looked at the event. The looking times recorded by the primary observer were used to determine when a trial had ended. Looking times during the initial and final phases of the familiarization and test trials were computed separately. Each trial ended when the infant either (1) looked away for 2 consecutive seconds after having looking for at least 2 cumulative seconds or (2) looked for 60 (familiarization, display) or 40 (test) cumulative seconds.

To assess inter-observer agreement, each trial was divided into 100-ms intervals, and the computer determined in each interval whether the two observers agreed on whether the infant was or was not looking at the event. Percent agreement was calculated for each trial by dividing the number of intervals in which the observers agreed by the total number of intervals in the trial. Agreement was measured for 45 of the 46 infants (only one observer was present for one infant) and averaged 95% per trial per infant.

Preliminary analyses of the test data revealed no significant interaction of condition and event with sex, *F*(2, 34)  = 2.37, *p*>.10; the data were therefore collapsed across sex in subsequent analyses.

### Results and Discussion

#### Familiarization trials

The infants’ looking times during the final phases of the four familiarization trials were averaged and compared by means of a 3×2 analysis of variance (ANOVA) with condition (different-, same- or no-word) and test event (old- or new-object) as between-subjects factors. No effect was significant, all *F*s <2.25, *p*>.11, suggesting that the infants in the six groups tended to look equally during the familiarization trials (different-word/new-object: *M* = 21.4, *SD* = 4.2; different-word/old-object: *M* = 26.2, *SD* = 12.4; same-word/new-object: *M* = 22.6, *SD* = 7.7; same-word/old-object: *M* = 23.9, *SD* = 9.1; no-word/new-object: *M* = 26.8, *SD* = 8.2; no-word/old-object: *M* = 19.5, *SD* = 6.0).

#### Display trial

The infants’ looking times during the display trial were analyzed as above. No effect was significant, all *F*s <1.29, *p*>.26, indicating that the infants in the six groups looked about equally during the display trial (different-word/new-object: *M* = 14.6, *SD* = 7.4; different-word/old-object: *M* = 15.8, *SD* = 8.9; same-word/new-object: *M* = 18.1, *SD* = 8.6; same-word/old-object: *M* = 13.9, *SD* = 9.4; no-word/new-object: *M* = 15.8, *SD* = 9.7; no-word/old-object: *M* = 10.5, *SD* = 4.8).

#### Test trial

**Figure 2:**
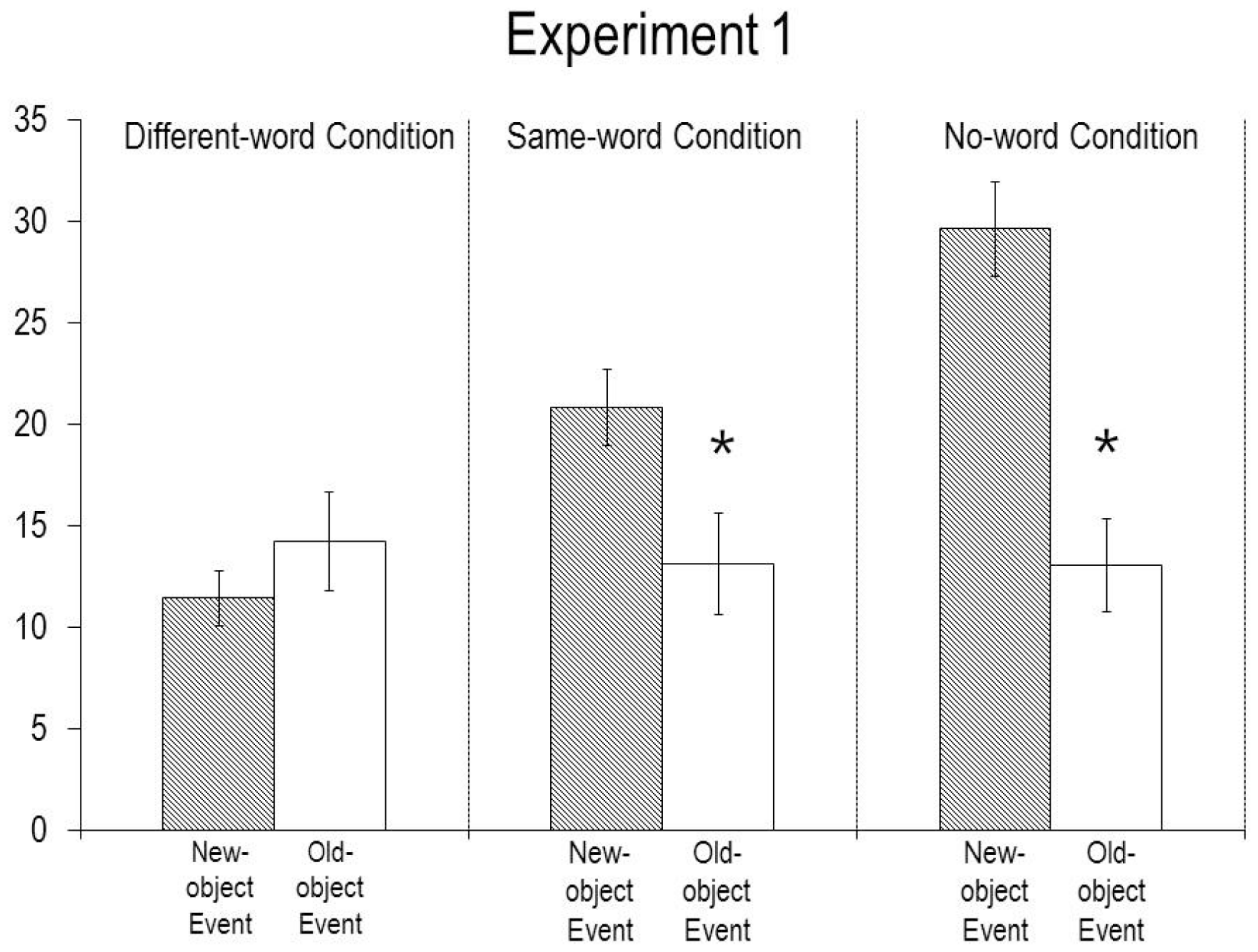
Mean looking times (s) of the infants in Experiment 1 during the test trial. Errors bars represent standard errors, and an asterisk denotes a significant difference between the events within a condition (p<.05 or better).

The infants’ looking times during the final phase of the test trial (see Fig. 2) were analyzed as above. The analysis yielded significant main effects of condition, *F*(2, 40)  = 8.02, *p*<.0025, and event, *F*(1, 40)  = 16.24, *p*<.001, as well as a significant Condition X Event interaction, *F*(2, 40)  = 10.47, *p*<.001. Planned comparisons revealed that (1) in the different-word condition, the infants who saw the new-object (*M* = 11.4, *SD* = 3.8) and old-object (*M* = 14.2, *SD* = 6.9) events looked about equally, *F*(1, 40) <1; (2) in the same-word condition, the infants who saw the new-object event (*M* = 20.8, *SD* = 5.0) looked reliably longer than those who saw the old-object event (*M* = 13.1, *SD* = 6.6), *F*(1, 40)  = 5.80, *p*<.025; and (3) in the no-word condition, the infants who saw the new-object event (*M* = 29.6, *SD* = 6.6) also looked reliably longer than those who saw the old-object event (*M* = 13.0, *SD* = 6.4), *F*(1, 40)  = 30.44, *p*<.0001. Wilcoxon rank-sum tests confirmed the results of the different-word (*W_S_* = 60.5, *p*>.40), same-word (*W_S_* = 37, *p*<.05), and no-word (*W_S_* = 38, *p*<.005) conditions.

An analysis of covariance (ANCOVA) showed that the same test results obtained after adjusting for the differences in the infants’ looking times during the familiarization and display trials. The analysis yielded significant main effects of condition, *F*(2, 38)  = 8.72, *p*<.0025, and event, *F*(1, 38)  = 16.39, *p*<.001, and a significant Condition X Event interaction, *F*(2, 38)  = 7.40, *p*<.0025.

Finally, comparisons focusing only on the new-object event showed that the infants in the different-word condition looked at this event reliably less than did those in the same-word condition, *F*(1, 13)  = 17.24, *p*<.0025, *W_S_* = 37.5, *p*<.0025, or in the no-word condition, *F*(1, 14)  = 45.13, *p*<.001, *W_S_* = 37, *p*<.0025. Thus, whereas the infants in the same- and no-word conditions viewed the new-object event as unexpected, the infants in the different-word condition did not. In contrast, comparisons focusing on the old-object event revealed no reliable differences among the three conditions, *F*(2, 20) <1, which thus differed only in their responses to the new-object event.

The infants in the no-word and same-word conditions looked reliably longer if shown the new- as opposed to the old-object event; in contrast, the infants in the different-word condition looked about equally at the two events. These divergent results make clear that the infants did not simply ignore the words they heard in each trial; but how did they interpret these words? It seems likely that during the familiarization trials the infants in the same-word and different-word conditions formed an expectation that the word “A dax!” communicated something about the agent’s subsequent action of obtaining the egg. When the infants in the same-word condition again heard “A dax!” at the start of the test trial, they no doubt expected the agent to reach for the egg as before, and they looked reliably longer when she did not. As for the infants in the different-word condition, one possible explanation for their responses, alluded to earlier, was that they (1) noticed the change in word at the start the test trial, (2) realized that this change in word might signal a change in the agent’s actions, but (3) were not able to immediately conjecture that the agent would now reach for the tree, as might an older child or an adult operating on a full-fledged mutual-exclusivity assumption. Because the infants lacked a specific expectation about what the agent would do next, they tended to look equally whether she reached for the egg or the tree. This interpretation, if valid, would suggest that by their first birthday, infants already realize that a change from one novel word to another may signal a change in action, even if they cannot go as far as to infer what this new action may be.

However, an alternative interpretation for the results of the different-word condition was that the infants were simply overwhelmed when they heard the new word (“A pilk!”). As a result, they could not process the test event further, and so they tended to look equally at the new- and old-object events. Experiment 2 tested this alternative interpretation.

## Experiment 2

In Experiment 2, 12-month-olds were tested in a different-word, a mixed-word, or a delayed-word condition. The different-word condition was identical to that in Experiment 1 except that all of the infants saw the new-object event in the test trial. Like the infants in the different-word condition, those in the mixed- and delayed-word conditions also saw the new-object event and heard the new word “A pilk!” in the test trial. As explained below, however, the infants in these conditions were expected to detect a violation in the new-object event and hence to look reliably longer at it than the infants in the different-word condition. We reasoned that such a positive result would rule out the notion that the infants in the different-word conditions of Experiments 1 and 2 responded as they did to the new-object event simply because they were overwhelmed by hearing the new word.

The infants in the *mixed-word* condition received familiarization and test trials identical to those in the different-word condition, with one exception: Two different novel words were used on alternate familiarization trials, “A dax!” and “A corp!”. If the infants in the different-word condition interpreted the word “A dax!” at the start of each familiarization trial as communicating something about the agent’s upcoming action on the egg and took the change in word at the start of the test trial to signal a possible change in the agent’s action, then the infants in the mixed-word condition might respond differently. Because the infants heard different words but saw the same actions across the four familiarization trials, they might be inclined to dismiss the words, which appeared to be unrelated to the agent’s actions, and they might rely solely on the agent’s actions (or choices) to reason about what she might do next. Thus, the infants should expect the agent to continue reaching for the egg in the test trial, regardless of the word uttered, and they should detect a violation when she reached for the tree instead.

The infants in the *delayed-word* condition received trials identical to those in the different-word condition, with one exception: In each familiarization and test trial, the agent grasped and lifted the toy *before*, rather than *after*, the word was uttered. If the infants in the different-word condition were interpreting the word uttered at the start of each familiarization trial as announcing something about the agent’s upcoming action on the egg, then the infants in the delayed-word condition, who heard the word only after the agent had already selected the egg, might respond differently. Specifically, they might attribute to the agent a preference for the egg, given her unvarying choices, and they might interpret the word “A dax!” as intended merely as a label for the egg. In the test trial, based on the prior-choice information from the familiarization trials, the infants should expect the agent to again reach for the egg, and they should detect a violation when she selected and labeled or commented on the tree instead. In short, we predicted that the infants in both the mixed- and delayed-word conditions would look reliably longer at the new-object event than the infants in the different-word condition.

### Methods

#### Participants

Participants were 24 healthy term infants, 12 male and 12 female (11 months, 7 days to 12 months, 29 days, *M* = 12 months, 2 days). Eight infants were randomly assigned to the different-word (*M* = 12 months, 0 day), mixed-word (*M* = 12 months, 3 days) or delayed-word (*M* = 12 months, 3 days) conditions. Another 16 infants were excluded because they looked for the maximum amount of time allowed in the test trial (6), were inattentive (3), fussy (3), or distracted (2), had a looking time in the test trial over 2.5 standard deviations from the mean of the condition (1), or experienced parental interference (1)

#### Apparatus and procedure

The apparatus and procedure in Experiment 2 were identical to those of the different-word condition in Experiment 1 with the following exceptions. First, all infants saw the new-object event in the test trial. Second, in the mixed-word condition, different novel words (“A dax!” and “A corp!”) were played in alternate familiarization trials; half the infants heard “A dax!” in the first and third trials and “A corp!” in the second and fourth trials, and half heard the two words in the reverse order. Finally, in the delayed-word condition, the events shown in the initial phase of each familiarization and test trial were re-ordered: The agent grasped and lifted the toy before, rather than after, the novel word was uttered twice. Inter-observer agreement was calculated for 23 of the 24 infants and averaged 96% per trial per infant. Preliminary analyses of the test data revealed no significant interaction between condition and sex, *F*(2, 18) <1; the data were therefore collapsed across sex in subsequent analyses.

### Results and Discussion

#### Familiarization trials

The infants’ looking times during the final phases of the four familiarization trials were averaged and compared by a single-factor ANOVA with condition (different-, mixed- or delayed-word) as a between-subjects factor. The main effect of condition was not significant, *F*(2, 21)  = 1.06, *p*>.36, indicating that the infants in the three conditions did not differ reliably in their mean looking times during the familiarization trials (different-word: *M* = 21.8, *SD* = 7.7; mixed-word: *M* = 30.0, *SD* = 13.7; delayed-word: *M* = 27.5, *SD* = 12.4).

#### Display trial

The infants’ looking times during the display trial were analyzed as above. The main effect of condition was again not significant, *F*(2, 21) <1, indicating that the infants in the three conditions tended to look equally during the display trial (different-word: *M* = 17.1, *SD* = 11.3; mixed-word: *M* = 14.9, *SD* = 8.2; delayed-word: *M* = 19.5, *SD* = 17.7).

#### Test trial

**Figure 3:**
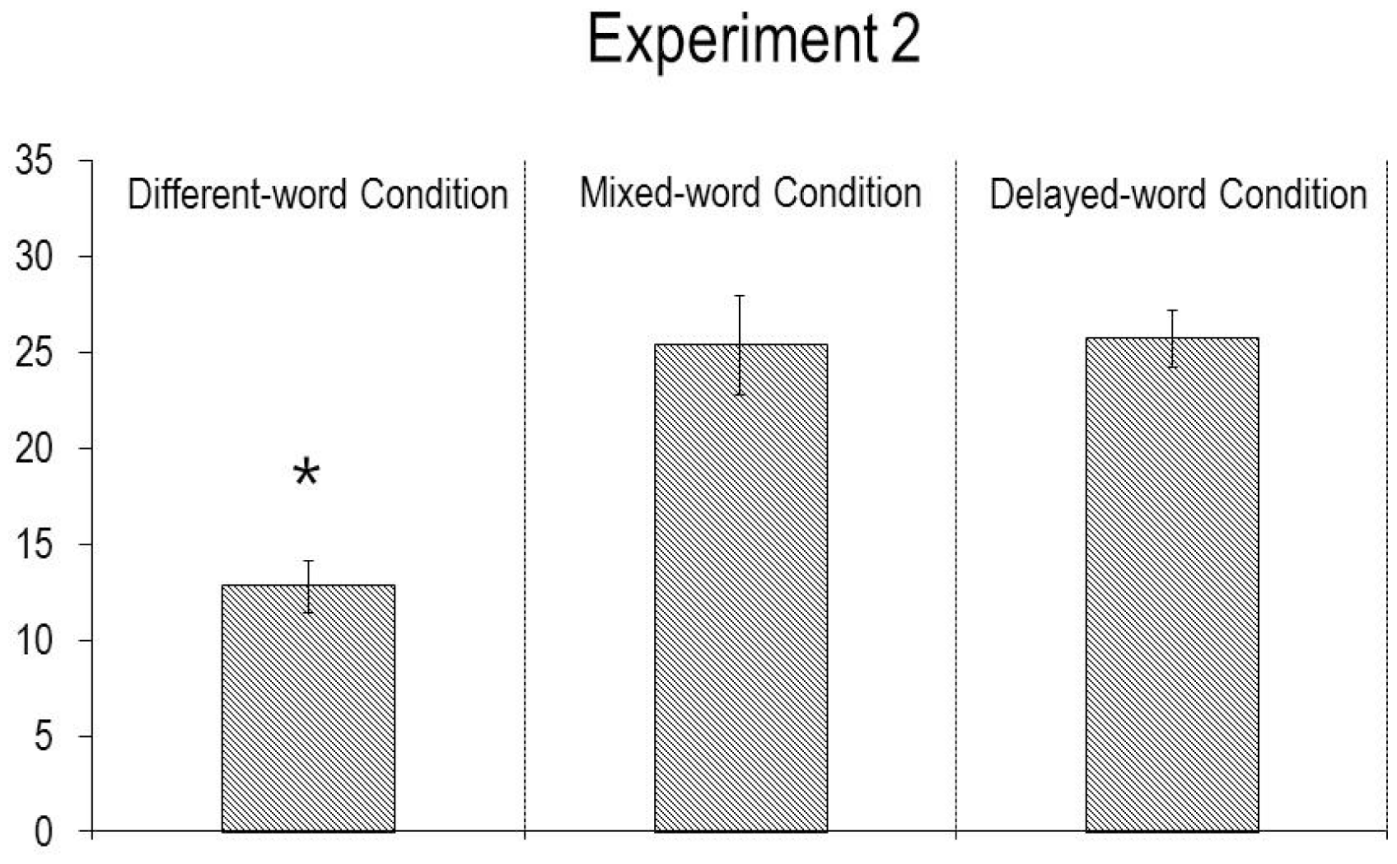
Mean looking times (s) of the infants in Experiment 2 during the test trial. Errors bars represent standard errors, and an asterisk denotes a significant difference between the conditions (p<.05 or better).

The infants’ looking times during the final phase of the test trial (see Fig. 3) were analyzed as above. The main effect of condition was significant, *F*(2, 21)  = 15.07, *p*<.0001, indicating that the infants’ looking times differed across conditions. An ANCOVA similar to that in Experiment 1 also revealed a significant main effect of condition, *F*(2, 19)  = 12.79, *p*<.001, and a non-parametric Kruskal-Wallis rank test confirmed this result, *H*(2)  = 14.47, *p*<.0025. A planned contrast revealed that, as expected, the infants in the mixed-word (*M* = 25.3, *SD* = 7.4) and delayed-word (*M* = 25.7, *SD* = 3.8) conditions looked reliably longer during the test trial than did the infants in the different-word condition (*M* = 12.8, *SD* = 4.2), *F*(1, 21)  = 23.24, *p*<.0001.

The results of Experiment 2 provide evidence against the notion that the infants in the different-word condition of Experiment 1 were simply overwhelmed when they heard the novel word “A pilk!” in the test trial. The infants in the mixed- and delayed-word conditions of Experiment 2 also heard the novel word “A pilk!” in the test trial, and yet they detected a violation when the agent grasped the tree. In the mixed-word condition, words and actions were not clearly related in the familiarization trials, and so the infants tended to dismiss the words and to focus on the actions; they attributed to the agent a preference for the egg, they expected her to maintain this preference in the test trial, and they detected a violation when she did not, as in prior preference tasks. In the delayed-word condition, the words in each trial arrived only after the agent had already grasped the object, as a label or comment on the object. Thus, at the start of the test trial, in order to predict what the agent would do, the infants could rely only on the agent’s prior actions in the familiarization trials: They had no other information to go on. Since these actions signaled a robust preference for the egg, the infants expected the agent to again grasp and verbalize about the egg, and they detected a violation when she performed these actions on the tree instead.

Together, the results of Experiments 1 and 2 thus support the possibility that the infants in the different-word conditions recognized that the change in word at the start of the test trial might signal a change in the agent’s upcoming actions, but were unable to form a specific expectation about what these new actions might be. We return to this possibility in the General Discussion.

## Experiment 3

The results of Experiments 1 and 2 indicated that, by 12 months of age, infants already show some sensitivity to a change in verbal information when reasoning about an agent’s actions in a simple two-object situation. At what age do infants begin to show such sensitivity? To begin to address this question, in Experiment 3 we tested 7-month-olds in *different-word* and *no-word* conditions identical to those in Experiment 1, with one main exception: We used a within-subject design, as is typically the case in preference experiments with younger infants [3], [4], [10], [17]. The infants received four test trials in which they saw the new- and old-object events on alternate trials, with order counterbalanced across infants in each condition.

In the no-word condition, we expected that the infants (1) would use the prior-choice information available in the familiarization trials to attribute to the agent a preference for the egg, (2) would expect the agent to maintain this preference in the test trials, and hence (3) would detect a violation when the agent reached for the tree in the new-object event. We thus predicted that the infants would look reliably longer at the new- than at the old-object event, in line with previous results with young infants [3], [4], [10].

Of greater interest was how the infants in the different-word condition would respond. At least two possibilities existed. On the one hand, the infants might attend to the verbal information provided and, like the 12-month-olds in the different-word condition of Experiment 1, look about equally at the new- and old-object events. On the other hand, the infants might simply ignore or dismiss the verbal information available, and like the infants in the no-word condition, look reliably longer at the new- than at the old-object event. We reasoned that this last pattern of results would provide additional evidence that the responses of the 12-month-olds in the different-word conditions of Experiments 1 and 2 reflect an *advance* in the development of infants’ sensitivity to verbal information when reasoning about others’ actions.

### Methods

#### Participants

Participants were 32 healthy term infants, 16 male and 16 female (7 months, 7 days to 8 months, 0 day, *M* = 7 months, 19 days). Another 4 infants were excluded because of distraction (1), fussiness (1), observer disagreements (1), or because the difference in the infant's looking times at the two test events was more than 3 standard deviations from the mean of the condition (1). Half the infants were randomly assigned to the different-word condition (*M* = 7 months, 20 days) and half to the no-word condition (*M* = 7 months, 19 days).

#### Apparatus and procedure

The apparatus and procedure in Experiment 3 were similar to those in Experiment 1, with two exceptions. First, as mentioned earlier, the infants saw the new- and old-object events on alternate trials for two pairs of test trials. Half the infants in each condition saw the new-object event first, and half saw the old-object event first. Second, slightly different criteria were used to end the display and test trials, to give the infants more time to process the events. In Experiment 3, the display and test trials now ended when the infant either (1) looked away for 2 consecutive seconds after having looking for at least 5 (display) or 3 (test) cumulative seconds, or (2) looked for 60 cumulative seconds. Inter-observer agreement averaged 95% per trial per infant. Preliminary analyses of the test data revealed no significant interaction of condition and event with sex or order, all *F*s <1.74, *p*>.20; the data were therefore collapsed across these latter two factors in subsequent analyses.

### Results and Discussion

#### Familiarization trials

The infants’ looking times during the final phases of the four familiarization trials were averaged and compared by a single-factor ANOVA, with condition (different-word or no-word) as a between-subjects factor. The main effect of condition was not significant, *F*(1, 30) <1, indicating that the infants in the two conditions did not differ reliably in their mean looking times during the familiarization trials (different-word: *M* = 26.0, *SD* = 12.0; no-word: *M* = 28.4, *SD* = 12.0).

#### Display trial

The infants’ looking times during the display trial were analyzed as above. The main effect of condition was not significant, *F*(1, 30) <1, indicating that the infants in the two conditions looked about equally during the display trial (different-word: *M* = 20.9, *SD* = 14.7; no-word: *M* = 23.5, *SD* = 16.6).

#### Test trials

**Figure 4:**
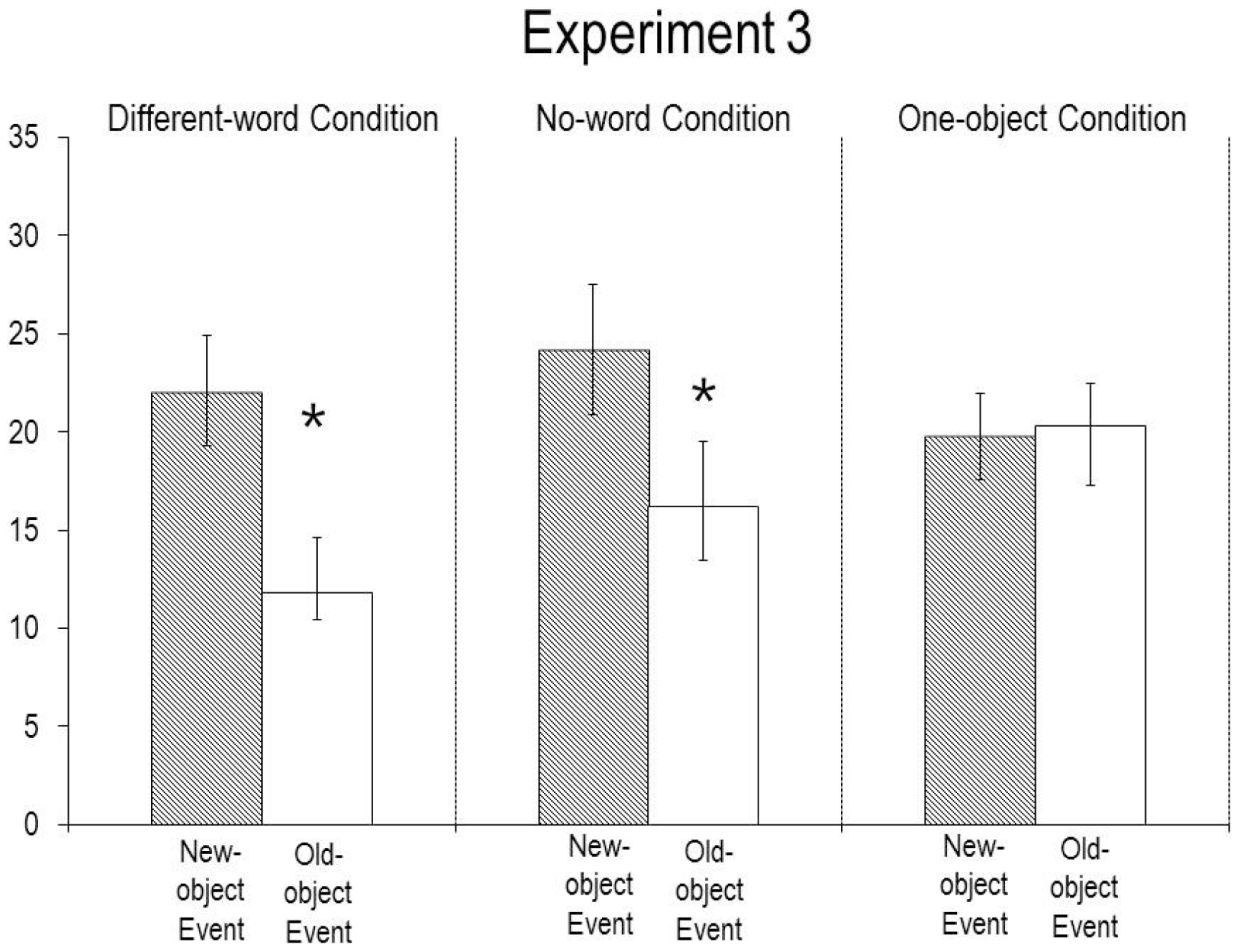
Mean looking times (s) of the infants in Experiment 3 during the test trials. Errors bars represent standard errors, and an asterisk denotes a significant difference between the events within a condition (p<.05 or better).

The infants’ looking times during the final phases of the four test trials (see Fig. 4) were averaged and compared by a 2×2 ANOVA with condition (different- or no-word) as a between-subjects factor and event (new- or old-object) as a within-subject factor. The main effect of event was significant, *F*(1,30)  = 38.29, *p*<.0001, indicating that the infants looked reliably longer overall at the new-object (*M* = 23.1, *SD* = 12.2) than at the old-object (*M* = 14.0, *SD* = 8.8) event. The main effect of condition was not significant, nor was the interaction between condition and event, both *F*s (1, 30) <1, suggesting that the same response pattern held in the two conditions. Planned comparisons confirmed that the infants in the different-word condition looked reliably longer at the new-object (*M* = 22.1, *SD* = 11.3) than at the old-object (*M* = 11.8, *SD* = 5.4) event, *F*(1, 30)  = 24.33, *p*<.0001, as did those in the no-word condition (new-object: *M* = 24.2, *SD* = 13.3; old-object: *M* = 16.2, *SD* = 11.0, *F*(1, 30)  = 14.58, *p*<.001). Inspection of the individual infants’ looking times revealed the same patterns: 15 of the 16 infants in the different-word condition (non-parametric Wilcoxon signed-ranks test, *T* = 3, *p*<.001), and 14 of the 16 infants in the no-word condition (*T* = 6, *p*<.0025), looked longer at the new- than at the old-object event.

#### First test trial analyses

Another ANOVA examined the infants’ responses in the first test trial they received; in this analysis, condition (different- or no-word) and event (new- or old-object) were between-subjects factors. These responses mirrored those found when the data from both pairs of test trials were considered. The analysis revealed a significant main effect of event, *F*(1, 28)  = 7.85, *p*<.01, indicating that the infants who saw the new-object event (*M* = 30.4, *SD* = 18.9) looked reliably longer overall than those who saw the old-object event (*M* = 14.6, *SD* = 13.0). The main effect of condition was not significant, *F*(1, 28)  = 2.91, *p* = .10, nor was the interaction between condition and event, *F*(1, 28) <1, suggesting that the same pattern held in both the different-word condition (new-object: *M* = 25.2, *SD* = 16.6, old-object: *M* = 10.1, *SD* = 4.1) and the no-word condition (new-object: *M* = 35.5, *SD* = 20.7, old-object: *M* = 19.1, *SD* = 17.3).

In additional analyses, we compared the first test trial looking times of the 7-month-olds in Experiment 3 to those of the 12-month-olds in Experiment 1. ANOVAs with age (7 or 12 months) and event (new- or old-object) as between-subjects factors were conducted separately for the no-word and different-word conditions. These analyses confirmed that the 12-month-olds in Experiment 1 and the 7-month-olds in Experiment 3 (1) responded similarly in the no-word condition, *F*(1, 28) <1 (at both ages, infants looked longer if shown the new- as opposed to the old-object event), but (2) responded differently in the different-word condition, *F*(1, 28)  = 7.32, *p*<.025 (the 7-month-olds looked longer if shown the new- as opposed to old-object event, whereas the 12-month-olds looked equally at both events).

#### Further results: One-object condition

The 7-month-olds in the different-word and no-word conditions of Experiment 3 looked reliably longer at the new- than at the old-object event, suggesting that they (1) used the choice information available in the familiarization trials to attribute to the agent a preference for the egg, (2) expected her to maintain this preference when the toys’ positions were switched, and thus (3) detected a violation in the new-object event when she reached for the tree. However, other interpretations were possible: Perhaps these young infants simply had a baseline preference for the new- over the old-object event, or perhaps they formed an association between the hand and the egg during the familiarization trials and looked longer at the new-object event during the test trials because it deviated from this association.

To test these alternative interpretations, additional 7-month-olds were tested in a *one-object* condition adapted from prior research [4], [17], [20], [34]. As was mentioned in the Introduction, when the non-target object is absent during the familiarization trials, infants have no basis for attributing to the agent a preference for the target object (e.g., she might reach for it simply because it is the only object available), and hence they tend to look equally at the new- and old-object events in the test trials. The one-object condition was thus identical to the no-word condition of Experiment 3 except that only the egg was present during the familiarization trials; following these trials, the egg was moved to the right side of the apparatus, the tree was added on the left, and the display and test trials proceeded as in the no-word condition. Participants were 16 infants, 8 male and 8 female (7 months, 7 days to 8 months, 0 day; *M* = 7 months, 19 days); no infant was excluded. A one-way ANOVA with event (new- or old-object) as a within-subject factor revealed that, as predicted, the infants tended to look equally at the new-object (*M* = 19.8, *SD* = 8.8) and old-object (*M* = 20.3, *SD* = 12.0) events, *F*(1, 30) <1. Of the 16 infants tested, only 8 looked longer at the new- than at the old-object event, *T* = 65, *p*>. 80. Additional ANOVAs revealed that the responses of the infants in the one-object condition differed reliably from those of the infants in the different-word (*F*(1, 30)  = 12.48, *p*<.0025) and no-word (*F*(1, 30)  = 8.24, *p*<.01) conditions of Experiment 3.

The results of Experiment 3 confirm prior findings that young infants can use prior-choice information to determine which of two objects an agent is likely to act on next [3], [4], [10], [17]. The infants in the different-word and no-word conditions expected the agent to reach for the egg in the test trials, based on her unvarying choices in the familiarization trials, and they therefore detected a violation when she reached for the tree instead. This expectation was not due to low-level factors: When only the egg was present in the familiarization trials, so that choice information was no longer available, the infants had no expectation about which object the agent would select in the test trials, in line with previous results from one-object tasks [4], [17], [20], [34].

## General Discussion

The present research examined whether infants could interpret a change in word as signaling a change in which of two objects an agent would reach for next. At 7 months, infants simply ignored the change in word; they expected the agent to continue reaching for the same object as before, and they looked reliably longer if the agent reached for the other object instead. At 12 months, infants realized that the change in word might signal a change in the agent’s actions, but they were unable to form the specific expectation that the agent would now reach for the other object; as a result, they tended to look equally whether the agent reached for the same object or for the other object.

The results with the 12-month-olds support two main conclusions. First, they confirm previous evidence (reviewed in the Introduction) that infants this age possess only a fledgling mutual-exclusivity assumption and, in particular, have difficulty using this assumption to determine the referents of novel words [24]–[28], [30], [31]. Upon hearing the different novel word “A pilk!” at the start of the test trial, the infants in the different-word conditions of Experiments 1 and 2 did not immediately conjecture—as an adult surely would have—that the agent would now reach for the other object on the apparatus floor, the tree. Nevertheless, the infants did glean some information from this change in word: They realized that it might signal a change in the agent’s actions. As such, our results indicate that 12-month-olds’ fragile mutual-exclusivity assumption enables them not only to individuate objects, as shown in prior research [24], but also to anticipate a possible change in an agent’s actions.

Second, and more generally, our results provide rich evidence that infants can flexibly attend to and integrate different types of information when reasoning about an agent’s actions in a simple two-object situation. When no verbal information was provided, the infants attributed to the agent a preference for the egg, based on her unvarying choices across the four familiarization trials, and they expected her to continue acting in accordance with this preference in the test trial (no-word condition, Experiment 1). When mixed words were spoken at the start of the familiarization trials (“A dax!”, “A corp!”), so that it was unclear whether or how these words related to the agent’s actions, the infants dismissed the words and focused on the actions; thus, they again attributed to the agent a preference for the egg, and they expected her to maintain this preference in the test trial (mixed-word condition, Experiment 2). When the same word accompanied the agent’s actions in each familiarization trial (“A dax!”), and this word was spoken *after* the agent grasped the egg, the infants interpreted the word simply as a label or comment on the egg; they used the evidence that the agent consistently chose the egg to attribute to her a preference for the egg, and they expected her to continue acting on this preference in the test trial (delayed-word condition, Experiment 2). When the same word accompanied the agent’s actions in each familiarization trial (“A dax!”), but this word was spoken *before* the agent grasped the egg, the infants apparently construed the word as a communication announcing that the agent would reach for the egg. When the infants heard the same word at the start of the test trial (“A dax!”), they expected the agent to again reach for the egg (same-word condition, Experiment 1); however, when the infants heard a different word at the start of the test trial (“A pilk!”), they realized that the agent’s actions might now change, even though they were unable to fathom what these new actions might be (different-word conditions of Experiments 1 and 2). Implicit in these findings is thus suggestive evidence that, depending on what verbal information was provided when, infants construed the agent’s actions differently: In some cases, they interpreted the agent’s actions as directed toward the goal of obtaining (and perhaps then commenting on) her preferred object; in other cases, they interpreted the agent’s actions as directed toward the goal of announcing which object she would reach for and then proceeding to do so. Thus, from an early age, and even before they can fully understand an agent’s communications, infants already attempt to integrate these communications into their interpretations of the agent’s actions.

The present findings suggest two directions for future research. One will be to determine at what age between 7 and 12 months infants in the different-word condition begin to show signs that they interpret the different word (“A pilk!”) as communicating a possible change in the agent’s upcoming actions. Given prior findings that 9-month-olds already demonstrate some sensitivity to mutual exclusivity in object-individuation tasks [25], we might perhaps expect 9-month-olds to respond like the 12-month-olds in Experiments 1 and 2, rather than like the 7-month-olds in Experiment 3.

Another research direction will be to examine at what age infants in the different-word condition successfully form the expectation, upon hearing the word “A pilk!” at the start of the test trial, that the agent will now reach for the other object on the apparatus floor, the tree. Given prior results that infants age 17–18 months and older can use mutual exclusivity to link novel words to their referents, at least under some conditions [35], we might perhaps expect 17- to 18-month-olds to look reliably longer in the test trial if shown the old- as opposed to the new-object event. Such a finding would not only provide additional evidence of infants’ developing ability to use mutual exclusivity, but it would reveal just how subtle and context-sensitive infants’ psychological interpretations can be: Specifically, when infants construe the agent’s actions as directed toward the goal of obtaining her preferred object, they detect a violation when she suddenly and inexplicably reaches for the other, non-preferred object; in contrast, when infants construe the agent’s actions as directed toward the goal of announcing which object she will reach for and then proceeding to do so, they detect a violation when she announces that she will now reach for the other object and yet reaches for the same object as before.

## Conclusion

Previous research indicates that when an agent consistently reaches for the same one of two distinct objects during familiarization trials, infants attribute to the agent a preference for this target object, they expect the agent to maintain this preference when the objects’ positions are switched, and they detect a violation when the agent, for no apparent reason, reaches for the non-target object instead. The present research indicates that 12-month-olds may interpret these very same actions differently depending on the verbal information that accompanies them. In particular, if infants hear the same word at the start of each familiarization trial, they assume that this word communicates that the agent will now reach for the target object; if this word changes, they realize that the agent’s actions may also change, and they no longer detect a violation when the agent reaches for the non-target object. Thus, as early as 12 months of age, the very same action—reaching for the non-target object—may be viewed as unexpected or as expected, depending on the communicative context in which it occurs.
